# Can repeated *in vivo* micro-CT irradiation during adolescence alter bone microstructure, histomorphometry and longitudinal growth in a rodent model?

**DOI:** 10.1371/journal.pone.0207323

**Published:** 2018-11-15

**Authors:** Tanvir Mustafy, Aurélie Benoit, Irène Londono, Florina Moldovan, Isabelle Villemure

**Affiliations:** 1 Department of Mechanical Engineering, École Polytechnique of Montréal, Station Centre-Ville, Montréal, Québec, Canada; 2 Sainte-Justine University Hospital Center, Montréal, Québec, Canada; 3 Department of Stomatology, Faculty of Dentistry, Université de Montréal, Montreal, Station Centre-Ville, Montréal, Québec, Canada; Indiana University Purdue University at Indianapolis, UNITED STATES

## Abstract

*In vivo* micro-computed tomography (micro-CT) can monitor longitudinal changes in bone mass and microstructure in small rodents but imposing high doses of radiation can damage the bone tissue. However, the effect of weekly micro-CT scanning during the adolescence on bone growth and architecture is still unknown. The right proximal tibia of male Sprague-Dawley rats randomized into three dose groups of 0.83, 1.65 and 2.47 Gy (n = 11/group) were CT scanned at weekly intervals from 4^th^ to 12^th^ week of age. The left tibia was used as a control and scanned only at the last time point. Bone marrow cells were investigated, bone growth rates and histomorphometric analyses were performed, and bone structural parameters were determined for both left and right tibiae. Radiation doses of 1.65 and 2.47 Gy affected bone marrow cells, heights of the proliferative and hypertrophic zones, and bone growth rates in the irradiated tibiae. For the 1.65 Gy group, irradiated tibiae resulted in lower BMD, Tb.Th, Tb.N and a higher Tb.Sp compared with the control tibiae. A decrease in BMD, BV/TV, Tb.Th, Tb.N and an increase in Tb.Sp were observed between the irradiated and control tibiae for the 2.47 Gy group. For cortical bone parameters, no effects were noticed for 1.65 and 0.83 Gy groups, but a lower Ct.Th was observed for 2.47 Gy group. Tibial bone development was adversely impacted and trabecular bone, together with bone marrow cells, were negatively affected by the 1.65 and 2.47 Gy radiation doses. Cortical bone microstructure was affected for 2.47 Gy group. However, bone development and morphometry were not affected for 0.83 Gy group. These findings can be used as a proof of concept for using the reasonable high-quality image acquisition under 0.83 Gy radiation doses during the adolescent period of rats without interfering with the bone development process.

## Introduction

*In vivo* micro-computed tomography (micro-CT) is an efficient tool for the non-destructive evaluation of laboratory animals and the *in vivo* tracking of longitudinal changes in bone mass and bone microstructure due to disease and/or bone adaptation processes [1, 2]. Micro-CT has emerged as an advancement from the simple X-ray imaging into an essential technique, which is now used for laboratory research, tissue engineering, and numerical modeling [3–6]. Micro-CT can be used to longitudinally monitor bone micro-architecture in growing animals at different developmental stages. It can provide animal specific high-resolution data of time-related changes in desired bone locations. Changes can result from pathological or therapeutic stimuli, assuming minimal or no effects of the micro-CT scanning radiations on the radiated bone structural system [7–9]. However, as the micro-CT system might impose relatively high ionizing radiation doses [10, 11], frequent or recurrent exposures to such doses of the scanned bony parts could induce some side effects, including growth hindrance, deformities of the skeleton, bone loss or other hematological abnormalities [7, 8, 12–14].

High-radiation doses scans provide better image sets, which further facilitate the assessment of trabecular and cortical bone structures with higher accuracy [9, 15, 16]. However, this dose increment might pose a risk to the normal bone development process. Bone tissue damage can occur with doses as low as 250 mGy [2, 17]. Cell death might occur due to the irreparable DNA damage resulting from excessive doses [9, 18]. Low radiation doses can also trigger the DNA damage checkpoint activation, which results in a decreased cell proliferation [19]. Hence, an effective approach must be established to acquire high-quality images while using minimal radiation exposure. This can be achieved by efficaciously optimizing the scanning parameters to produce a low radiation dose which will provide an acceptable image quality without affecting the bone tissue.

Different studies use different approaches to investigate bone structure. Some studies need a single micro-CT scan whereas some need repeated CT scans. The impact of single radiation dose on longitudinal bone growth has been extensively investigated. Human long bones can exhibit swelling and fragmentation symptoms for doses ranging from 3–5 Gy [8]. Also, it has been reported that a radiation dose in the order of 5 Gy can affect the bone regeneration process while a dose limit of 2.5 Gy showed no such impacts [20]. A rabbit femur exposed to 3.5 Gy radiation dose showed a significant reduction in the growth of long bones [21], whereas no adverse effects were noticed for 400 mGy and lower radiation doses on the proliferation and differentiation of osteoblasts in adult Sprague-Dawley rats [22].

Repeated micro-CT measurements deemed to be necessary especially when tracking changes in bone development. Repeated measurements can provide valuable information on bone quality in post-surgical scenarios or in response to physical exercise or pharmaceutical treatment. However, repeated CT-scans can also cause a threat to the bone if it crosses a safe limit. Numerous animal studies have been performed to assess the impact of repeated micro-CT radiation doses on the whole body or the exposed limb. In a recent study [23], repeated (4 scans) doses effects of 1255 mGy and 453 mGy were investigated in adult mice (17 weeks old) femurs and no effects were found. In another study [24], adult Wister rats (30 weeks old) underwent 8 weeks *in vivo* scanning on their right tibia using doses as high as 939 mGy per scan. Bone structural measurements remained unaffected under the applied scanning regime. Another study [25] used adult mice aged 12 week old (exposed to 845.9 mGy) and adult rats aged 8 months old (exposed to 596.6 mGy) and found a decrease in the trabecular bone volume fraction in the radiated tibiae compared to the control ones.

Both single and repeated radiation studies demonstrate that various animal protocols showed divergent adaptability for the level of radiation doses applied. In addition to the difference in experimental protocols, variances in animal size, shape and anatomy, which put the skeleton under thicker or thinner skin, could be partly responsible for such differences in response to radiations. Hence, radiation results from one animal model and protocol could not be directly extrapolated to another. Moreover, most of the radiation doses related studies were performed on adult animal models, where the bone tissue has already peaked to its skeletal maturity. However, no such studies have been performed to define limit values below which radiation doses can be used safely for a growing animal model, in which bones have not reached their skeletal maturity.

Thus, the objective of this study was to evaluate radiation effects on bone morphometry, bone marrow cells, bone growth rate and growth plate histomorphometry in growing tibiae for three radiation doses from repeated *in vivo* micro-CT scanning in adolescent rats. Results of this study will provide knowledge on weekly radiation doses protocol which can provide high-quality image sets to adequately investigate trabecular and cortical compartments, without causing damage to bone development during the rat adolescent growing period. The present study covered the rat adolescent period, which spans from the beginning of the 4^th^ week of age to the end of 12^th^ week period [26], resulting in a 9-week scanning period to investigate the radiation doses effects by comparing the irradiated and non-radiated limbs. The radiation dose of the first group was set at 0.83 Gy/scan, evaluated as the baseline to produce reasonable image quality for bone development investigation purpose. Two-fold (1.65 Gy/scan) and three-fold (2.47 Gy/scan) dose values were tested along with the same protocols for the second and third radiation groups.

## Materials and methods

### Animals

21 days old male Sprague-Dawley rats (n = 33) were obtained from Charles River Laboratories, Montreal, Canada. Rats were randomly divided into three doses groups: 0.83 Gy, 1.65 Gy and 2.47 Gy (n = 11 per group). They were given 1-week of acclimatization before starting the experiment. Rats were housed two and three per cage (dimension 53 × 35.5 cm) at 25°C with a 12:12-hour light-dark cycle and provided with a standard laboratory diet and water ad libitum. Body weight was monitored weekly. The experimental protocol and all animal procedures were carried out in accordance with the guidelines of the Canadian Council on Animal Care (CCAC) and were approved by the Institutional Animal Care Committee at the Research Center of Sainte-Justine University Hospital, Montreal, Canada.

### Repeated micro-CT scanning

**Fig 1:**
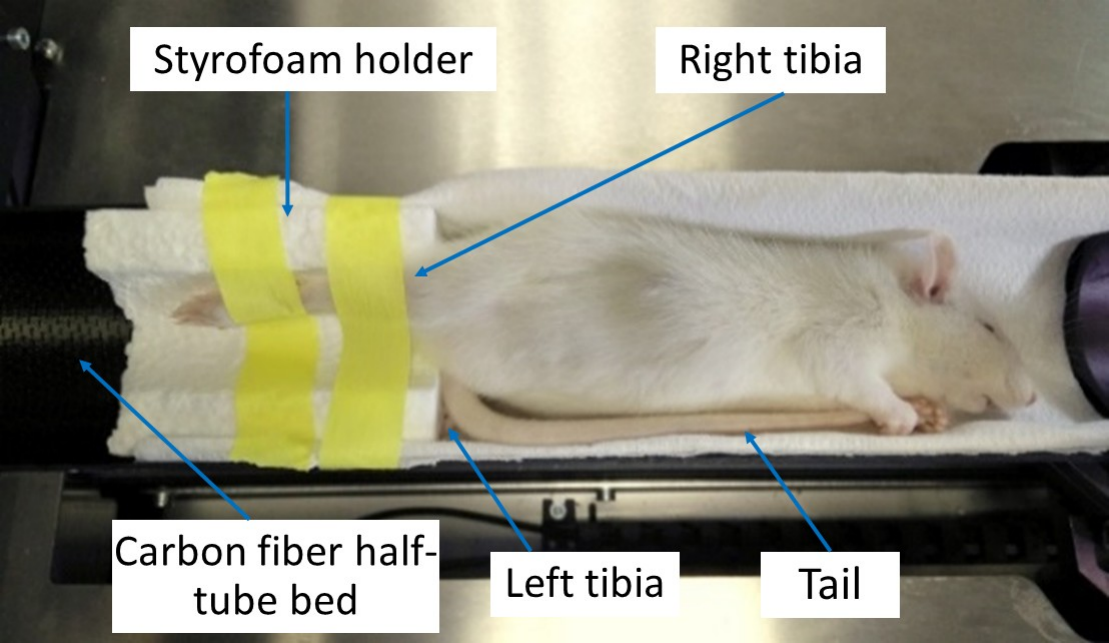
Rat positioning on the Skyscan 1176 scanner for *in vivo* scanning. The rat was placed sideways on the scanning bed while kept anesthetized (anesthesia mask not shown). This configuration was adapted to facilitate the positioning of the irradiated leg (right) into the iso-center of the scanning chamber. The right tibia was secured into a Styrofoam holder (1 cm thick) of cylindrical shape and firmly held with a medical adhesive tape. The non-radiated leg (left) was folded towards the animal’s head and placed alongside the animal with its tail.

A micro-CT scanner was used to perform nine weekly basis repeated CT scans of the proximal right tibia of the rats from their 4^th^ to 12^th^ weeks of age. A final scan was performed at the 14^th^ week. The two-week interval for the last scan was chosen to assess the maximal radiation exposure effect after the end of the exposure protocol [27, 28]. The imaging system was a Skyscan 1176 *in-vivo* micro-CT (Skyscan, N.V., Belgium) scanner with rotatable X-ray source and detector. Each rat was anesthetized (2% isoflurane, 1.0 L/min O_2_) and maintained on anesthetic gasses for the duration of the scanning. The rat was secured in the carbon fiber half-tube bed of the Skyscan 1176, and the right tibia was positioned into a Styrofoam holder of cylindrical shape. This procedure was performed to place the rat tibia in the scanning midline of the scanner and to eliminate any unwanted movement of the tibia during the radiation period. (Fig 1) [29]. The left tibia together with the tail were folded towards the animal’s head and placed alongside the animal on the carbon fiber half-tube bed using masking tape. An ophthalmic gel was applied to the eyes of the rat during the entire scanning period to prevent dryness. The radiated tibia was subjected to X-rays solely, without irradiating the contralateral limb (left tibia).

For the first radiation group (0.83 Gy), all scans were performed on the anesthetized rats with an isotropic voxel size of 18μm. The choice of 18μm was made based on the previous findings [30, 31], which enables a reasonable high-quality image for the trabecular and cortical bone tissue investigation. An overview of image acquisition and reconstruction parameters for three radiation groups have been given in a tabular format (Table 1). For 0.83 Gy group, the isotropic voxel size generated 1,336×1,680 CCD detector array. Total irradiation time was 5 min 34 sec and the scanning consisted of a stack of 304 images. For the second radiation group (1.65 Gy), image acquisition parameters were similar to the first group except for an improvement in the additional frame averaging (2 frame averaging versus 1 frame averaging) (Table 1). This improvement resulted in a finer detector array compared to the previous one (1,336×2,000 CCD detector array versus 1,336×1,680 CCD detector array). Total irradiation time was 11 min 9 sec and the scanning consisted of a stack of 395 images. For the third radiation group (2.47 Gy), an isotropic voxel size of 9μm was chosen for acquiring high-quality image sets for assessing trabecular and cortical bone microarchitecture. Image acquisition parameters were similar to the first group (Table 1). However, due to the improvement in isotropic voxel size (9μm versus 18μm), a finer detector array was generated compared to the first group (2,672×3,560 CCD detector array versus 1,336×1,680 CCD detector array). Total irradiation time was 16 min 39 sec and the scanning consisted of a stack of 304 images.

**Table 1:** Image acquisition and reconstruction parameters of the rat proximal tibiae for the three doses groups.

	0.83 Gy	1.65 Gy	2.47 Gy
***Scanning parameters***			
Voxel size (μm)	18	18	9
Voltage (kV)	65	65	65
Current (μA)	385	385	385
Rotation step (over 180°)	0.65°	0.50°	0.65°
Exposure time (ms)	350	350	1140
Frames averaged per projection	1	2	1
Filter	AL 1mm	AL 1mm	AL 1mm
Approximate scan time (min)	6	11	17
***Reconstruction Parameters***			
Filter	Gaussian	Gaussian	Gaussian
Smoothing kernel	1	1	1
Ring artifact reduction	4	4	4
Beam hardening correction (%)	10	10	10
Attenuation coefficient	0.000–0.049	0.000–0.049	0.000–0.049
***Analysis Parameters***			
Thresholding	Global, 65	Global, 65	Global, 65

All micro-CT scans were obtained using the SkyScan 1176 model, Bruker-microCT.

For all groups, the left tibia was used as a control and scanned only on the last (14^th^ week) scanning time point. Euthanasia of the rats was performed after the last scan (14^th^ weeks of age) using a CO_2_ chamber. For all rats, weight monitoring was conducted on a weekly basis to assess the impact of anesthesia and irradiation on rat development. The acquisition covered the proximal tibial section of the rat tibia. The delivered doses of 0.83 Gy, 1.65 Gy and 2.47 Gy computed tomography dose index (CTDI) were calculated based on the manufacturer specifications (Bruker micro-CT). The provided specifications (Bruker micro-CT) followed the dose measurements using a UNFORS PS-2 patient skin dosimeter. Shielding was provided with acrylic plastic (PMA) tubes of various wall thicknesses to simulate soft biological tissue. Local absorbed radiation dose rate (mGy/min) for tibia, femur, etc. have been provided by the manufacturer for different scan settings scenarios [32]. The data that accurately matched with our scanning parameters (65 kV, 385 μA, full x-ray, and 1-mm Al filter) have been extracted and local absorbed dose rate have been multiplied by the scanning time to get the resulted doses for our study [33] (S3 File). For an approximation, the tissue at all depths was assumed to be a cylinder and the dose rate of all tissue cylinder diameters averaged between the dose in the air (zero depth) and the dose at the cylinder center (half diameter) [32].

Scanned image sets were reconstructed by applying filtered back-projection algorithm (software NRecon, v.1.6.10, Skyscan, Kontich, Belgium) [29]. A total height of 10 mm cross-sectional images was reconstructed for every scanned set. The reconstruction started from the beginning of the knee joint and extended distally into the tibial diaphysis. The resolution of the processed images for first and second radiation groups was 1500 × 1500 pixels each, 17.48 μm isotropic voxel size, and the images were 8-bit in size (256 gray levels). The third radiation group produced images with 2700 × 2700 pixels each, 8.74 μm isotropic voxel size, and the images were 8-bit in size (256 gray levels).

### Calcein injections

For measurement of longitudinal bone growth rate, calcein was used to label the bone line on the surface of the tibia. Injections of calcein (Sigma-Aldrich, St. Louis, MO, USA), a fluorescent marker, were made intraperitoneally at a dosage of 15 mg/Kg [34]. Injections were done 5 and 2 days prior to euthanasia.

### Bone marrow cell assessment

After CO_2_ asphyxiation, followed by decapitation, both tibiae were collected. Left (control) and right (irradiated) tibiae were sawed off to keep 10 mm on both proximal and distal sides using an ISOMET 1000 Precision Saw (Buehler, An ITW Company, Illinois, USA). To determine cell radiation damage, bone marrow cells were collected from both control and radiated tibiae. Bone marrow cells were flushed by applying pressure with a needle filled with HBSS (Hank's balanced saline solution) in the sawed part of the tibiae. A cell count was performed on the collected cell suspensions on HBSS with trypan blue (0.4% solution, Sigma-Aldrich, Oakville, ON, Canada). Using the trypan blue test, the number of living and dead cells and their corresponding percentages were determined for both control and radiated tibiae. From these values, percentage of unaffected bone marrow cells was calculated by dividing the total number of live cells by the number of total cells (live + dead).

### Tissue processing

Formalin solution (Anachemia, Montreal, QC, Canada) was used to fix the proximal sections (~10 mm) from each tibia for a duration of 48h. Thereafter, graded alcohol solutions were used for dehydration, xylene was used for clarification and methylmethacrylate (MMA) (Fisher Scientific Canada, Nepean, ON, Canada) was used for embedding process [35]. When the polymerization was completed, a microtome (Leica SM2500) setup was used to cut the blocks of the tibiae into 6 μm sections. Only the proximal sections were used in this study. To cover the 40–50% of the growth plate depth, the tibiae were cut along the longitudinal bone axis for 36 slides, six series of six slides, which contain two sections per slide. To facilitate the growth rate measurements, the first slide of each series (6 slides, 12 sections total) per proximal tibia were set aside from light. A microscope (Leica DMR with Retina Qimaging Camera) was used for slice observation while using 5x magnification for growth rate measurements.

### Bone growth rate

**Fig 2:**
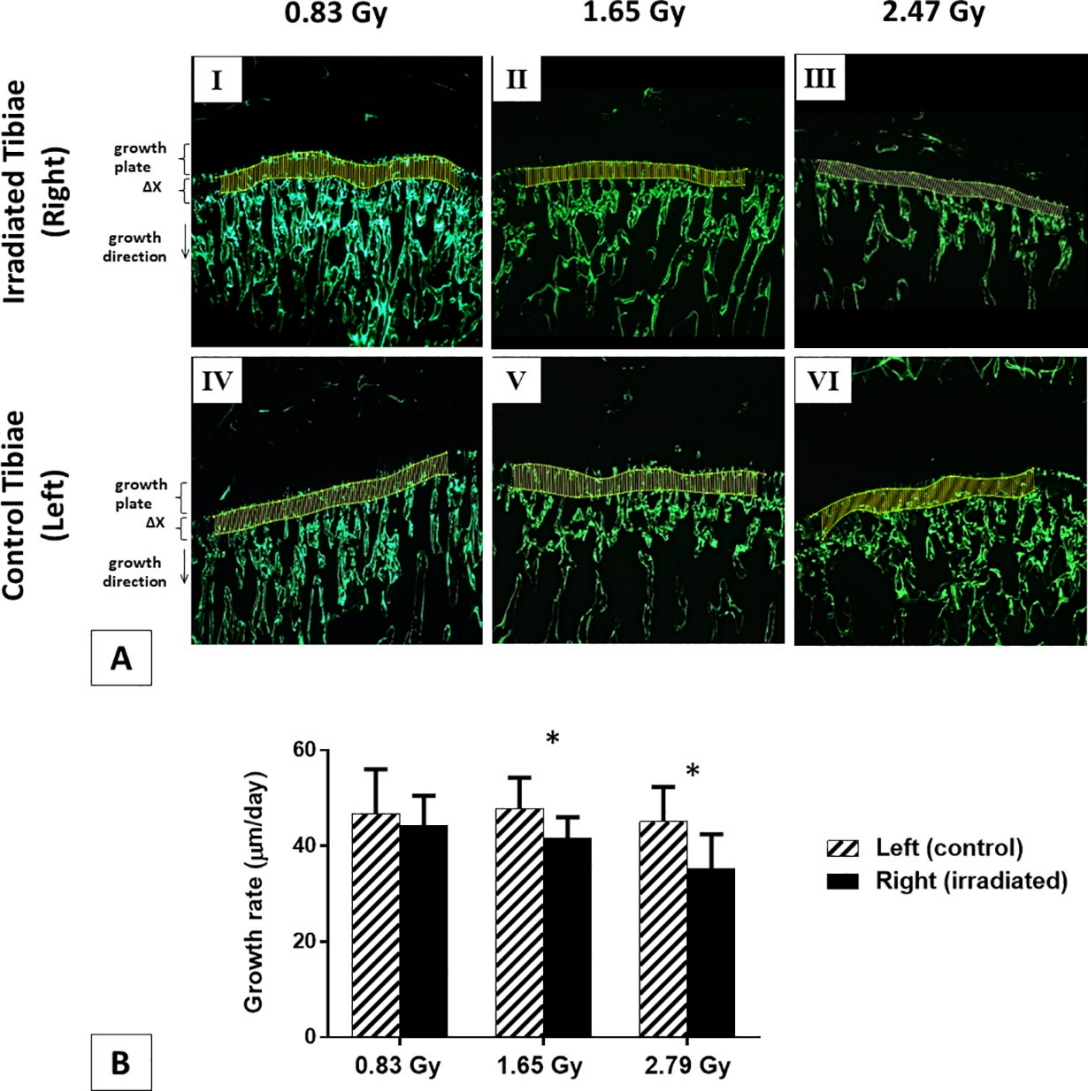
Bone growth rates (μm/day) measurements. (A) 5x magnified microscopic images of the tibial metaphysis labeled twice with calcein for representative irradiated and control tibiae from three doses groups (Ⅰ-ⅤⅠ). Bone growth (ΔX, μm) measured as the mean distance between the two calcein lines, which were modeled as splines and divided by the time interval (3 days) between the two applied injections. (B) Growth rates (μm/day) of rat proximal tibiae for 0.83, 1.65 and 2.47 Gy radiation groups (mean value ± SD). *: a significant difference (*p* < 0.05) between the control (left) and irradiated (right) tibiae for each radiation dose.

The distance between two calcein labels was divided by the time interval (3 days) between the two applied injections to calculate the bone growth rate [36]. An in-house built Matlab program was used for this purpose. The distance was automatically calculated as the mean value of 100 segments parallel to the longitudinal growth direction with both calcein lines modeled as splines [34, 35] (Fig 2A).

### Growth plate histomorphometry

**Fig 3:**
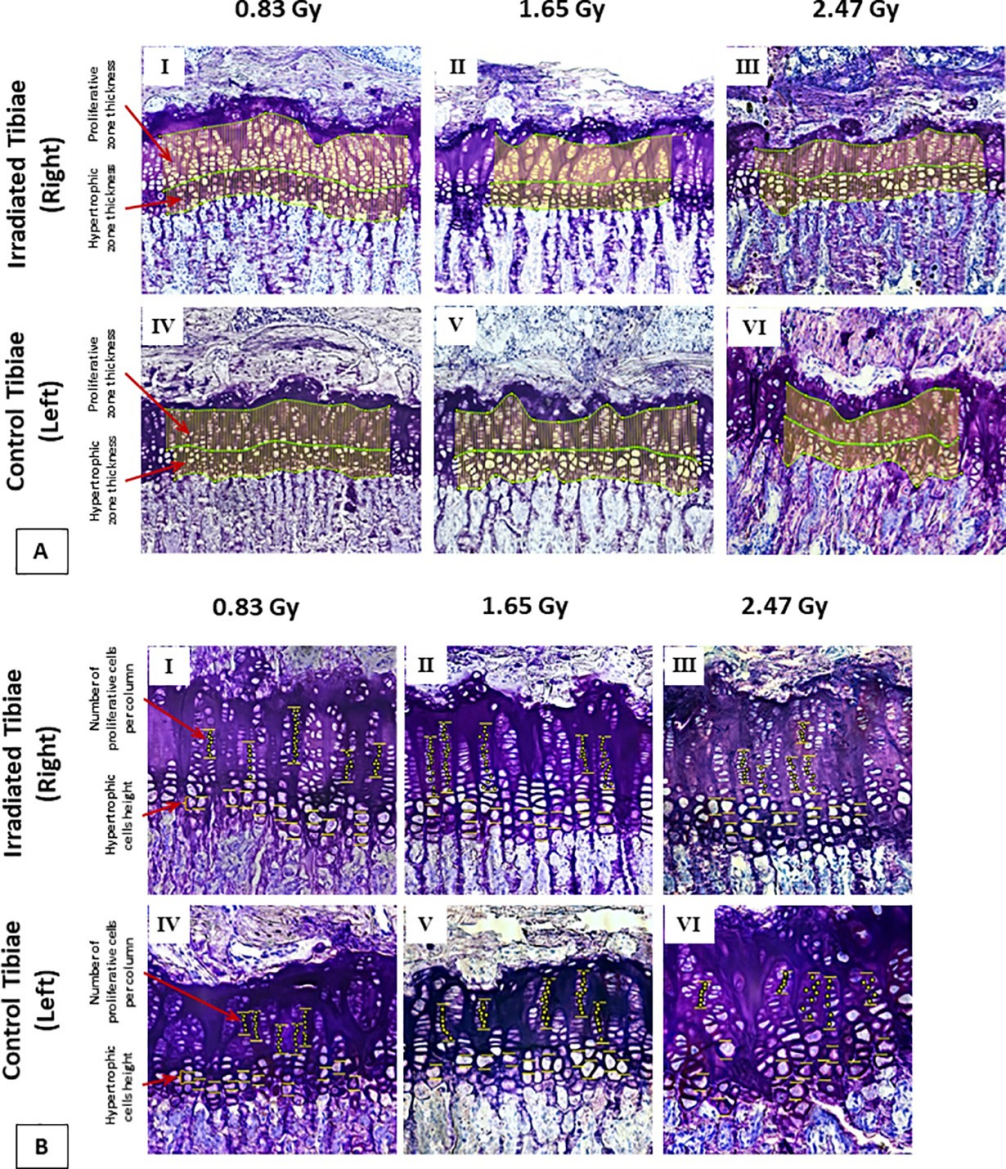
Histomorphometry measurement. (A) Growth plate section embedded in MMA and stained with toluidine blue (10x). Evaluation of the hypertrophic and proliferative zonal thicknesses for three doses groups. (B) Growth plate section embedded in MMA and stained with toluidine blue (20x). Evaluation of the hypertrophic cell height and number of proliferative cells per column for three doses groups.

Heights of the proliferative and hypertrophic zones, the hypertrophic cell height as well as the number of proliferative cells per column were measured for the histomorphometric analysis, similarly to previous work [34, 35] (Fig 3A and 3B). Hypertrophic cell height and the number of proliferative chondrocytes per column were measured as they are considered to be the indirect markers of bone growth [34, 35]. To measure heights, a similar approach to the bone growth rate measurements was implemented with 10x magnified image sets. Values from 100 segmental measurements were averaged for the assessment of zonal heights (Fig 3A). A 20x magnified image set was used to measure the hypertrophic cell height along the longitudinal growth direction (Fig 3B). The number of proliferative chondrocytes per column was measured from 20x magnified image sets for six random columns per growth plate (Fig 3B). For a single proximal tibial segment, histomorphometric parameters were measured by averaging 72 values, 6 values per section, 12 values per microscope slide with a six series repetition.

### Trabecular and cortical bone morphometry

**Fig 4:**
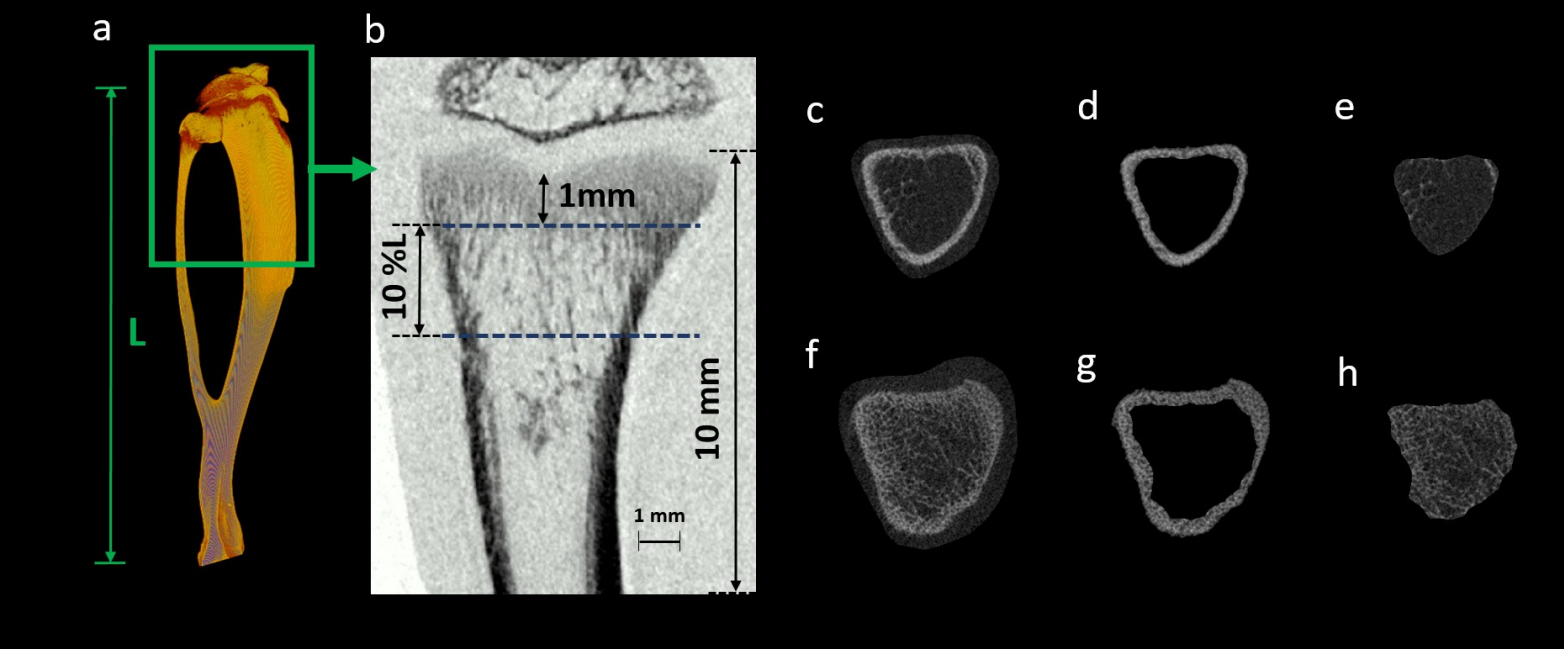
*In vivo* scanning of proximal tibia and bone segmentation process. (a) A representative 3D reconstructed tibia showing the total tibial length (L). (b) Scanned proximal tibial cross-section (10 mm in height) of the rat tibia. This representative image was acquired from a 17.48-μm pixel size scanning at 0.83 Gy radiation dose. VOI consisting trabecular and cortical bone, for morphometric parameters evaluation, beginning at ~1mm distal to the growth plate and extending for 10% of the total tibial length (L). Proximal (f) and distal (c) tibial sections are illustrated. The cortical (d, g) and trabecular (e, h) bone regions were segmented using a semi-automatic bone segmentation algorithm.

A volume of interest (VOI) was defined for morphometric analysis from the reconstructed image sets. The VOI included the proximal metaphysis, covering both trabecular and cortical bony segments (software CT Analyzer v.1.13, Skyscan, Kontich, Belgium). The proximal metaphysis of the tibia contains the growth plate and is responsible for blood supply and vascular stasis in growing bone. This part is also very sensitive to radiation exposure compared to the other regions of the bone [37]. So, absence of radiation effects on this bony region could presumably be considered as to have no effects on the epiphysis and the metaphysis parts as well [24]. The VOI was selected as a percentage of the entire tibial length (L) to keep consistency with the growing tibial length from 4^th^ to 14^th^ week of age. To exclude the primary spongiosa, the VOI started at ~1mm distal to the growth plate and extended for 10% of the total tibial length (L) [38] (Fig 4).

An in-house algorithm was developed for semi-automatically segmenting the trabecular and cortical bone. The segmentation was done by delineating the periosteum and endosteum surface in a semi-automatic algorithm based approach [39, 40]. A global gray threshold value of 65 corresponding to an equivalent density of 0.413 g/cm^3^ of calcium hydroxyapatite (CaHA), was set for all the analysis [38, 39]. Morphometric analysis was performed using CTAn software v.1.13 for the selected VOI of trabecular bone to evaluate the following bone structural parameters: bone mineral density (BMD), bone volume fraction (BV/TV), connectivity density (Conn.Dn), trabecular number (Tb.N), trabecular thickness (Tb.Th), and trabecular spacing (Tb.Sp) [41]. Excluding the VOI of trabecular bone from the selected dataset, cortical bone VOI was also extracted. Cortical microarchitectural measurements, including tissue mineral density (TMD), cross-sectional area inside the periosteal envelope (Tt.Ar), cortical bone area (Ct.Ar), cortical thickness (Ct.Th), periosteum perimeter (Ps.Pm), endocortical perimeter (Ec.Pm), medullary area (Ma.Ar), and mean eccentricity (Ecc) were evaluated using the cortical bone VOI [41].

The morphometric measurement process was appraised for reproducibility test. To do so, five scans of the right tibia were acquired from a dead rat in different orientations. After the completion of each scan, the rat was completely removed from the scanner bed and repositioned again in a different orientation. The same micro-CT scanning, image reconstruction, VOI selection and morphometric analysis protocols as the ones used for the radiation effects experiment were used in this reproducibility evaluation. The coefficient of variation (CV) was then determined by the five scans. The resulting reproducibility was high, with CV found to be less than 2% for BV/TV, Ct. Th., Ec. Pm., and Ma. Ar., less than 3% for BMD, TMD, Tb.Th, Tb.N, Tt. Ar., Ps. Pm., and Ecc., and less than 4% for Ct. Ar., Tb.Sp, and Conn.Dn.

### Statistical analysis

Statistical analyses were performed using SPSS Statistics (v. 23, IBM). Comparisons were made at the 14^th^ week between the irradiated and non-radiated tibiae for impacts on bone marrow cells, bone growth rate, growth plate histomorphometry, and bone morphometry for each dose group [42]. ANOVA test (general linear model) was performed to determine time effects, radiation dose, and their interaction on body weight. A paired Student’s t-test was performed for determining any significant differences in absolute and percentage numbers of viable cells, in average bone growth rates and in histomorphometric and bone structural parameters measured at the 14^th^ week for both irradiated and control tibiae. Moreover, structural properties of trabecular and cortical bone microstructure of the irradiated tibiae from three doses groups were statistically analyzed on 14^th^ week scanning data. A one-way ANOVA with Tukey’s multiple comparisons was performed to assess the significant group difference and pairwise comparisons. For each group, the series mean value was used to replace any values which were missing due to the movement of rats during a scanning procedure or due to the reconstruction error. For all the groups, this missing value incident occurred a total of five times (once in the 0.83 Gy group at 8^th^ week of age, twice in the 1.65 Gy group at 6^th^ and 9^th^ week of age, and twice in the 2.47 Gy group at 7^th^ and 11^th^ week of age). Results were considered statistically significant for *p* < 0.05.

## Results

### Bone growth rate

The average bone growth rate measured at the 14^th^ week in irradiated tibiae for 1.65 and 2.47 Gy group resulted in growth rate reductions of 13.1% and 21.8% respectively with respect to the control tibiae. These reductions were statistically significant (*p* < 0.05) (Fig 2B). No significant difference was observed for the bone growth rate in the 0.83 Gy group (Fig 2B).

### Growth plate histomorphometry

**Fig 5:**
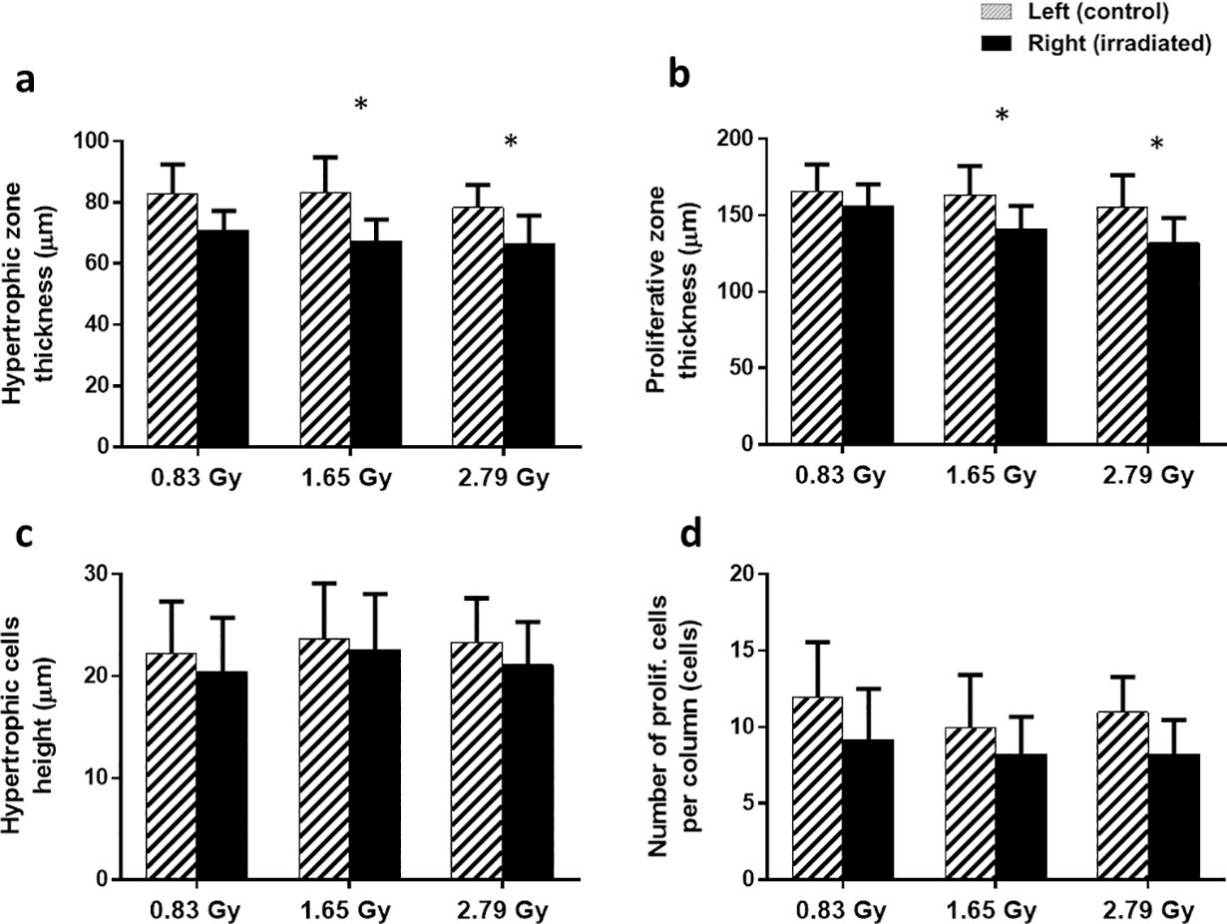
Histomorphometry measurements comparison for control and irradiated tibiae. (a-d) Growth plate histomorphometry measurements of rat proximal tibiae for 0.83, 1.65 and 2.47 Gy radiation groups (mean value ± SD). *: a significant difference (*p* < 0.05) between the control (left) and irradiated (right) tibiae for each radiation dose.

Significant differences were found in the zone thickness for both HZ and PZ in 1.65 and 2.47 Gy groups, whereas no significant difference was found for the 0.83 Gy group between the irradiated and control tibiae (Fig 5A and 5B). Hypertrophic cell heights and numbers of proliferative chondrocytes per column were found to be similar for irradiated and non-radiated tibiae for all three groups (Fig 5C and 5D).

#### Trabecular and cortical bone morphometry: Comparative analysis at the 14^th^ week

**Fig 6:**
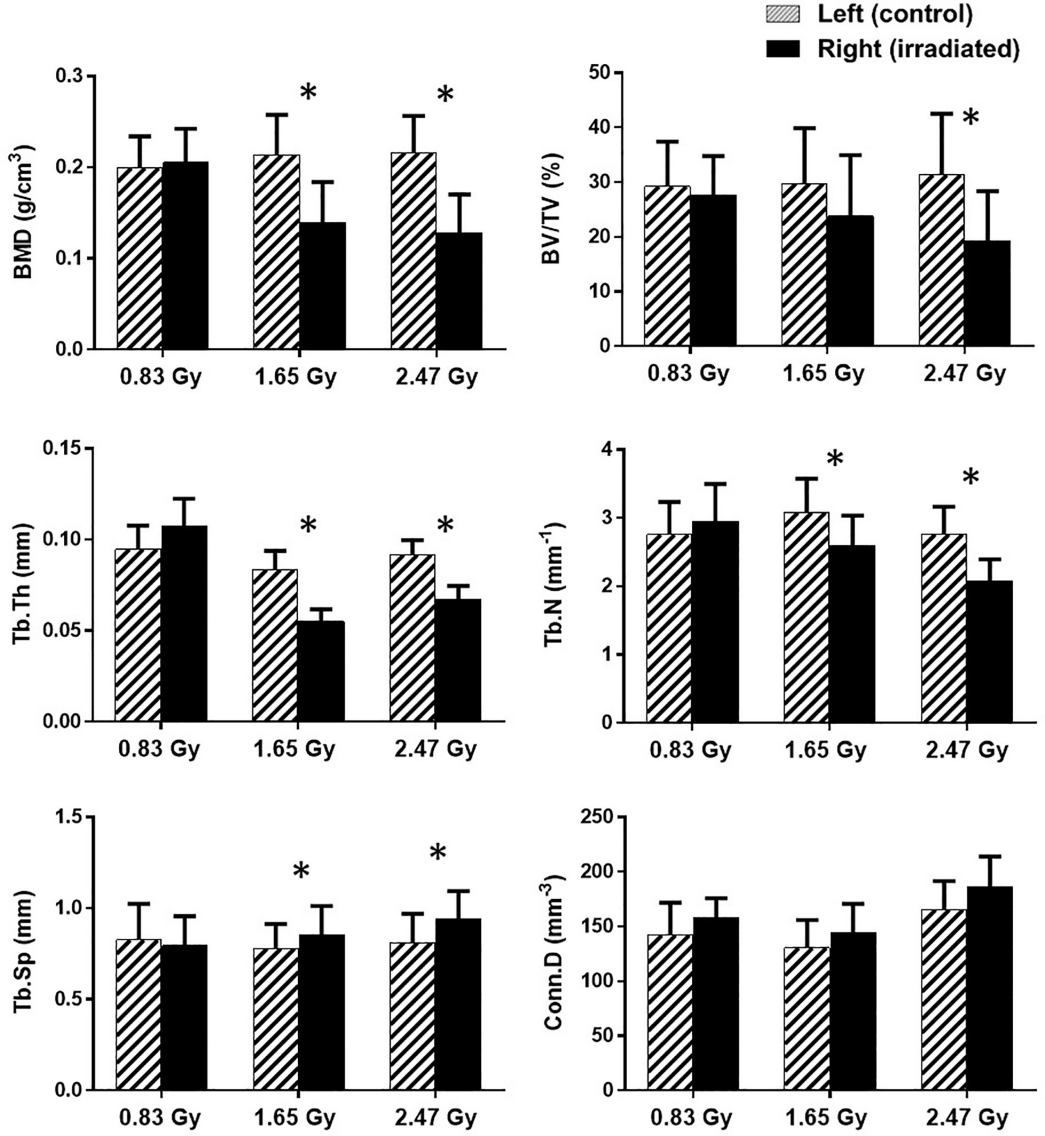
**Mean values and standard deviations of the trabecular bone parameters for the left (hatched columns), and right tibia (black columns) at 14^th^ week of age (n = 11/group).** *: a significant difference (*p* < 0.05) between the control (left) and irradiated (right) tibiae for each radiation dose.

The effect of repeated *in vivo* irradiation was assessed by comparing the repeatedly irradiated right tibiae to the singly irradiated left tibiae at the 14^th^ week of age. Morphometric parameters of both trabecular and cortical bones were compared within each group to assess the radiation effect. For the trabecular bone morphometry, 0.83 Gy group showed no significant difference between the irradiated tibiae and their contralateral controls (Fig 6). For both the 1.65 and 2.47 Gy group, a significant decrease in BMD, Tb.Th, Tb.N, and a significant increase for Tb.Sp was observed between the irradiated and control tibiae (Fig 6). Moreover, a significant decrease in BV/TV was also observed for the 2.47 Gy group (Fig 6).

**Fig 7:**
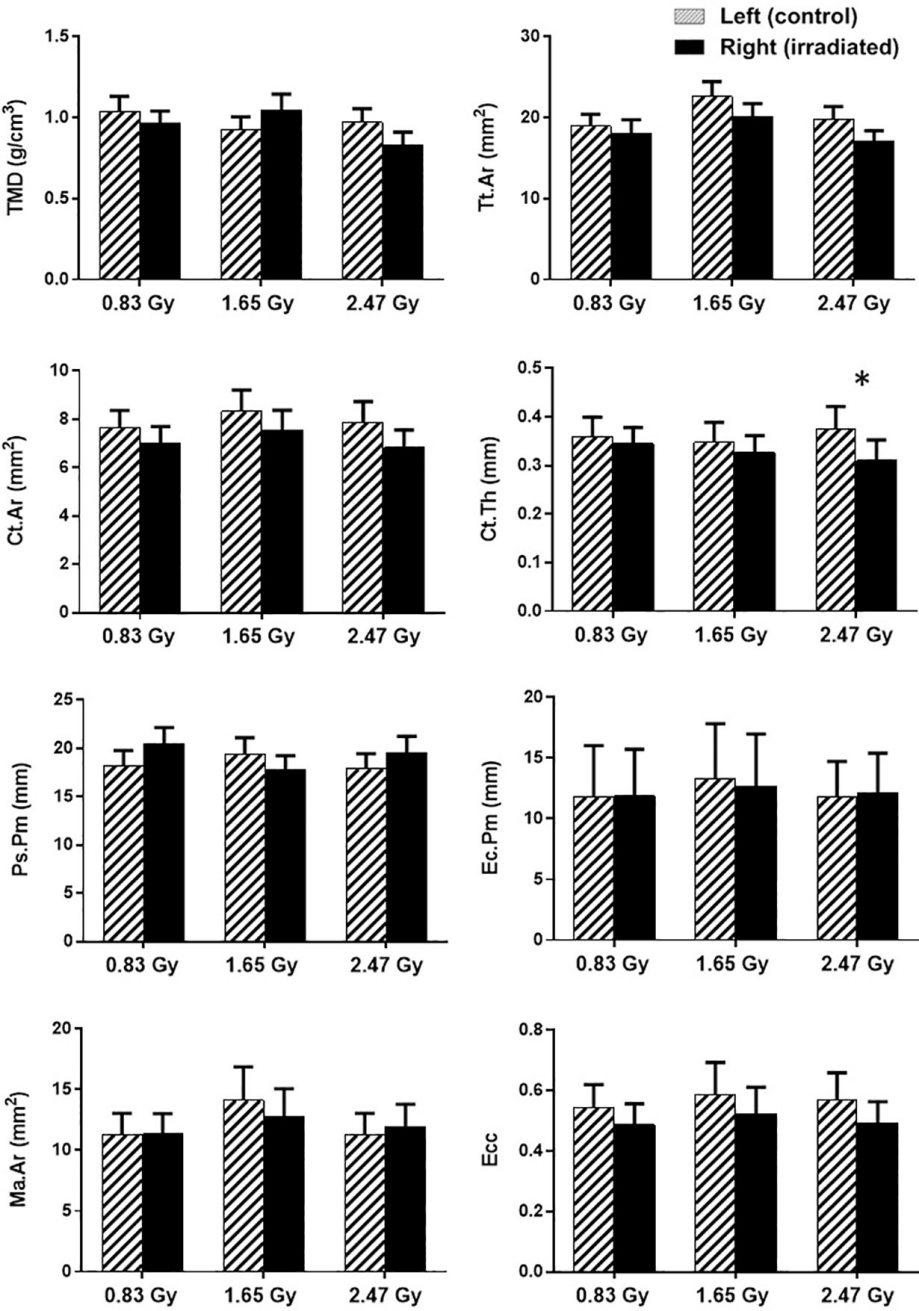
**Mean values and standard deviations of the cortical bone parameters for the left (hatched columns), and right tibiae (black columns) at 14^th^ week of age (n = 11/group).** *: a significant difference (*p* < 0.05) between the control (left) and irradiated (right) tibiae for each radiation dose.

For the cortical bone morphometry, no differences were found between the irradiated and control tibiae at the 14th week of age for both 0.83 and 1.65 Gy group (Fig 7). However, irradiated tibiae resulted in lower Ct.Th compared to the controlled ones for the 2.47 Gy group (Fig 7).

### Trabecular and cortical bone morphometry: 9-week longitudinal comparative analysis

Bone morphometric changes were assessed in the right proximal tibia during the entire adolescent period (from 4^th^ to 14^th^ week of age) for each rat. Trabecular bone parameters showed changes with rat development in the different groups. For the 0.83 Gy group, a significant increase (*p* < 0.05) was observed for BMD, BV/TV, Tb.Th, and Tb.N from the 4^th^ to the 14^th^ week old period (Table 2). However, a decrease was observed for Conn.Dn values within the same study period (Table 2). A significant increase for Tb.Sp and a decrease for Tb.Th were observed for both 1.65 Gy and 2.47 Gy group (Table 2). However, an increase in Tb.N for 1.65 Gy and a decrease in Conn.Dn and BMD were observed for 1.65 Gy and 2.47 Gy group respectively (Table 2). A significant increase (*p* < 0.05) was observed for Tt.Ar, Ct.Ar, Ps.Pm, Ec.Pm, Ma.Ar, and Ecc for all three groups (Table 3). However, for TMD and Ct.Th values, a significant increase was only observed for 0.83 Gy and 1.65 Gy groups (Table 3).

**Table 2:** Longitudinal assessment of trabecular microarchitecture of the right proximal tibial metaphysis in three doses groups of rats.

*Trabecular structural properties*	*Dose (Gy)*	*Age (week)*									
		4	5	6	7	8	9	10	11	12	14
***BMD (g.cm^-3^)***											
	*0*.*83*	0.156 ± 0.035	0.163 ± 0.027	0.150 ± 0.024	0.171 ± 0.029	0.183 ± 0.04	0.172 ± 0.051	0.179 ± 0.044	0.185 ± 0.026	0.200 ± 0.031	0.205 ± 0.037 ^**α**^
	*1*.*65*	0.162 ± 0.028	0.173 ± 0.046	0.163 ± 0.038	0.179 ± 0.041	0.121 ± 0.031	0.131 ± 0.016	0.131 ± 0.02	0.111 ± 0.036	0.125 ± 0.035	0.139 ± 0.045
	*2*.*47*	0.173 ± 0.015	0.192 ± 0.045	0.184 ± 0.029	0.186 ± 0.035	0.141 ± 0.051	0.14 ± 0.018	0.121 ± 0.051	0.121 ± 0.036	0.132 ± 0.034	0.128 ± 0.042 ^**γ**^
***BV/TV (%)***	* *										
	*0*.*83*	18.78 ± 5.23	20.05 ± 4.12	21.89 ± 6.42	22.27 ± 7.13	20.72 ± 4.13	21.07 ± 3.12	19.94 ± 6.14	21.51 ± 7.12	22.78 ± 5.12	27.56 ± 7.21 ^**α**^
	*1*.*65*	20.55 ± 8.23	21.52 ± 7.4	22.72 ± 8.24	23.77 ± 5.13	19.74 ± 8.23	19.77 ± 5.13	19.34 ± 8.13	22.3 ± 6.12	21.46 ± 7.24	23.76 ± 11.23
	*2*.*47*	22.47 ± 10.12	23.63 ± 8.23	20.12 ± 7.40	21.23 ± 6.40	18.75 ± 8.24	19.34 ± 7.23	18.84 ± 5.34	19.44 ± 6.14	17.76 ± 8.42	19.14 ± 9.23
***Tb.Th (mm)***	* *										
	*0*.*83*	0.078 ± 0.012	0.083 ± 0.012	0.081 ± 0.012	0.086 ± 0.013	0.087 ± 0.012	0.079 ± 0.011	0.084 ± 0.012	0.085 ± 0.015	0.087 ± 0.015	0.107 ± 0.015 ^**α**^
	*1*.*65*	0.093 ± 0.014	0.094 ± 0.011	0.086 ± 0.010	0.078 ± 0.010	0.075 ± 0.010	0.074 ± 0.012	0.076 ± 0.010	0.072 ± 0.012	0.062 ± 0.013	0.055 ± 0.006 ^**β**^
	*2*.*47*	0.103 ± 0.011	0.105 ± 0.011	0.097 ± 0.012	0.089 ± 0.015	0.093 ± 0.011	0.094 ± 0.011	0.088 ± 0.012	0.078 ± 0.011	0.076 ± 0.011	0.067 ± 0.007 ^**γ**^
***Tb.N (mm^-1^)***	* *										
	*0*.*83*	2.208 ± 0.457	2.104 ± 0.435	2.453 ± 0.508	2.578 ± 0.533	2.473 ± 0.482	2.447 ± 0.477	2.642 ± 0.515	2.577 ± 0.483	2.82 ± 0.529	2.946 ± 0.552 ^**α**^
	*1*.*65*	1.972 ± 0.379	1.863 ± 0.358	2.15 ± 0.414	2.12 ± 0.408	2.234 ± 0.414	2.163 ± 0.401	2.22 ± 0.411	2.41 ± 0.402	2.33 ± 0.388	2.601 ± 0.434 ^**β**^
	*2*.*47*	2.651 ± 0.496	2.55 ± 0.477	2.678 ± 0.501	2.489 ± 0.466	2.102 ± 0.359	1.961 ± 0.335	2.16 ± 0.369	2.1 ± 0.326	1.83 ± 0.284	2.072 ± 0.321 ^**γ**^
***Tb.Sp (mm)***	* *										
	*0*.*83*	0.839 ± 0.187	0.817 ± 0.182	0.784 ± 0.174	0.763 ± 0.159	0.819 ± 0.17	0.71 ± 0.151	0.766 ± 0.163	0.751 ± 0.153	0.85 ± 0.173	0.794 ± 0.162
	*1*.*65*	0.646 ± 0.133	0.612 ± 0.126	0.671 ± 0.138	0.726 ± 0.14	0.752 ± 0.146	0.781 ± 0.154	0.751 ± 0.148	0.795 ± 0.145	0.831 ± 0.152	0.855 ± 0.156 ^**β**^
	*2*.*47*	0.752 ± 0.131	0.723 ± 0.126	0.723 ± 0.126	0.711 ± 0.124	0.777 ± 0.135	0.795 ± 0.134	0.743 ± 0.125	0.773 ± 0.126	0.892 ± 0.145	0.929 ± 0.151 ^**γ**^
***Conn.Dn (mm^-3^)***	* *										
	*0*.*83*	189.8 ± 32.5	195.5 ± 39.1	234.7 ± 46.9	188.4 ± 32.6	178.5 ± 30.9	189.3 ± 32.8	234.1 ± 34.5	173.2 ± 25.5	164.3 ± 24.2	157.9 ± 29.2 ^**α**^
	*1*.*65*	263.4 ± 40.9	258.2 ± 40.1	273.8 ± 42.5	295 ± 54.2	219.5 ± 40.3	232.6 ± 42.7	266.4 ± 45.2	145.3 ± 24.6	132.1 ± 22.4	144.7 ± 26.1 ^**β**^
	*2*.*47*	208.8 ± 30.3	220.2 ± 31.9	255.2 ± 37	210.2 ± 30.5	233.2 ± 43.2	189.2 ± 35.1	225.8 ± 41.8	205.2 ± 27.7	178.3 ± 24.1	186.6 ± 27.4

Values are expressed as Mean ± SD, n = 11/group. Within each dose (0.83, 1.65 and 2.47 Gy), different symbols (**α**, **β**, **γ**) denote statistical significance (***p* < 0.05**) from 4^th^ to 14^th^ week of age. Abbreviations: BMD, bone mineral density; BV/TV, bone volume fraction; Tb.Th, trabecular thickness; Tb.N, trabecular number; Tb.Sp, trabecular spacing; Conn.Dn, connectivity density.

**Table 3:** Longitudinal assessment of cortical microarchitecture of the right proximal tibial metaphysis in three doses groups of rats.

*Cortical structural properties*	Dose (Gy)	*Age (week)*									
		4	5	6	7	8	9	10	11	12	14
***TMD (gm*.*cm*^*-3*^*)***											
	*0*.*83*	0.773 ± 0.063	0.803 ± 0.065	0.828 ± 0.08	0.873 ± 0.085	0.824 ± 0.08	0.858 ± 0.084	0.831 ± 0.082	0.852 ± 0.084	0.884 ± 0.087	0.961 ± 0.078 ^**α**^
	*1*.*65*	0.743 ± 0.08	0.783 ± 0.085	0.782 ± 0.085	0.823 ± 0.078	0.852 ± 0.08	0.802 ± 0.076	0.783 ± 0.074	0.883 ± 0.102	0.902 ± 0.104	1.043 ± 0.101 ^**β**^
	*2*.*47*	0.718 ± 0.069	0.744 ± 0.072	0.752 ± 0.069	0.783 ± 0.071	0.802 ± 0.073	0.783 ± 0.09	0.802 ± 0.092	0.773 ± 0.089	0.794 ± 0.092	0.83 ± 0.08
***Tt*.*Ar (mm*^*2*^*)***											
	*0*.*83*	10.54 ± 1.04	12.05 ± 1.19	15.42 ± 1.52	17.73 ± 1.81	18.6 ± 1.9	19.41 ± 1.99	19.84 ± 2.03	20.07 ± 0.17	20.45 ± 0.18	18.08 ± 1.46 ^**α**^
	*1*.*65*	11.75 ± 1.11	14.23 ± 1.35	18.23 ± 1.73	18.07 ± 1.45	19.69 ± 1.58	20.38 ± 1.63	20.52 ± 2.15	19.19 ± 2.01	18.89 ± 1.98	20.05 ± 1.78 ^**β**^
	*2*.*47*	10.1 ± 0.99	11.04 ± 1.09	13.37 ± 1.32	14.88 ± 1.31	16.34 ± 1.44	17.34 ± 1.52	17.58 ± 1.88	16.23 ± 1.73	16.34 ± 1.74	17.03 ± 1.34 ^**γ**^
***Ct*.*Ar (mm*^*2*^*)***											
	*0*.*83*	3.289 ± 0.356	4.586 ± 0.496	5.53 ± 0.599	5.132 ± 0.556	5.808 ± 0.592	5.884 ± 0.6	6.542 ± 0.667	7.261 ± 0.74	7.469 ± 0.762	7.013 ± 0.685 ^**α**^
	*1*.*65*	2.96 ± 0.302	4.62 ± 0.472	6.002 ± 0.614	5.994 ± 0.711	6.424 ± 0.762	7.32 ± 0.869	6.784 ± 0.867	7.728 ± 0.988	7.685 ± 0.982	7.55 ± 0.817 ^**β**^
	*2*.*47*	2.835 ± 0.321	4.05 ± 0.459	4.735 ± 0.536	4.23 ± 0.479	5.323 ± 0.649	6.23 ± 0.759	5.954 ± 0.726	6.34 ± 0.773	6.67 ± 0.952	6.823 ± 0.732 ^**γ**^
***Ct*.*Th (mm)***											
	*0*.*83*	0.196 ± 0.02	0.251 ± 0.026	0.235 ± 0.024	0.281 ± 0.029	0.288 ± 0.03	0.335 ± 0.035	0.364 ± 0.036	0.383 ± 0.037	0.373 ± 0.036	0.344 ± 0.033 ^**α**^
	*1*.*65*	0.232 ± 0.024	0.244 ± 0.025	0.215 ± 0.022	0.242 ± 0.028	0.263 ± 0.031	0.272 ± 0.032	0.312 ± 0.037	0.353 ± 0.042	0.325 ± 0.039	0.326 ± 0.035 ^**β**^
	*2*.*47*	0.22 ± 0.031	0.223 ± 0.031	0.18 ± 0.025	0.212 ± 0.034	0.249 ± 0.04	0.224 ± 0.036	0.258 ± 0.041	0.263 ± 0.051	0.281 ± 0.055	0.309 ± 0.042
***Ps*.*Pm (mm)***											
	*0*.*83*	13.48 ± 1.29	13.5 ± 1.29	15.45 ± 1.48	18.68 ± 2.24	19.23 ± 2.3	19.19 ± 2.3	17.23 ± 1.86	19.71 ± 2.12	19.78 ± 2.13	20.48 ± 1.64 ^**α**^
	*1*.*65*	11.96 ± 1.22	12.67 ± 1.29	16.18 ± 1.65	15.43 ± 1.76	18.08 ± 2.06	18.8 ± 2.14	18.57 ± 2.56	16.4 ± 2.26	18 ± 2.48	17.72 ± 1.51 ^**β**^
	*2*.*47*	12.67 ± 1.52	14.22 ± 1.71	16.34 ± 1.96	16.34 ± 1.77	18.75 ± 2.03	19.34 ± 2.09	18.84 ± 1.73	17.89 ± 1.65	18.63 ± 1.72	19.49 ± 1.73 ^**γ**^
***Ec*.*Pm (mm)***											
	*0*.*83*	9.51 ± 2.96	9.68 ± 3.01	11.15 ± 3.47	12.58 ± 4.62	12.68 ± 4.66	13.03 ± 4.79	12.92 ± 3.82	12.68 ± 3.75	12.77 ± 3.77	11.88 ± 3.84 ^**α**^
	*1*.*65*	10.45 ± 3.35	10.98 ± 3.52	12.39 ± 3.97	12.32 ± 4.27	12.91 ± 4.48	12.81 ± 4.44	13.13 ± 3.85	12 ± 3.51	11.86 ± 3.47	12.66 ± 4.32 ^**β**^
	*2*.*47*	9.54 ± 2.68	9.37 ± 2.64	10.42 ± 2.93	11.57 ± 4	11.76 ± 4.07	11.81 ± 4.08	12.08 ± 3.81	11.14 ± 3.51	11.02 ± 3.47	12.18 ± 3.23 ^**γ**^
***Ma*.*Ar (mm*^*2*^*)***											
	*0*.*83*	7.25 ± 1.37	7.46 ± 1.41	9.89 ± 1.87	12.59 ± 2.14	12.79 ± 2.17	13.52 ± 2.3	13.3 ± 2.18	12.81 ± 2.1	12.98 ± 2.13	11.34 ± 1.67 ^**α**^
	*1*.*65*	8.79 ± 1.86	9.6 ± 2.03	12.23 ± 2.59	12.08 ± 2.36	13.26 ± 2.59	13.06 ± 2.55	13.73 ± 2.58	11.46 ± 2.15	11.2 ± 2.11	12.81 ± 2.26 ^**β**^
	*2*.*47*	7.27 ± 1.39	6.99 ± 1.33	8.64 ± 1.65	10.65 ± 1.86	11.01 ± 1.92	11.11 ± 1.94	11.62 ± 1.93	9.89 ± 1.64	9.67 ± 1.61	11.91 ± 1.88 ^**γ**^
***Ecc***											
	*0*.*83*	0.383 ± 0.07	0.372 ± 0.068	0.437 ± 0.08	0.482 ± 0.082	0.473 ± 0.081	0.525 ± 0.09	0.539 ± 0.081	0.569 ± 0.086	0.582 ± 0.087	0.486 ± 0.069 ^**α**^
	*1*.*65*	0.421 ± 0.098	0.442 ± 0.103	0.483 ± 0.113	0.534 ± 0.109	0.505 ± 0.103	0.539 ± 0.11	0.528 ± 0.09	0.613 ± 0.105	0.627 ± 0.107	0.523 ± 0.087 ^**β**^
	*2*.*47*	0.341 ± 0.065	0.362 ± 0.069	0.382 ± 0.073	0.44 ± 0.075	0.442 ± 0.075	0.473 ± 0.081	0.55 ± 0.083	0.512 ± 0.078	0.522 ± 0.079	0.492 ± 0.07 ^**γ**^

Values are expressed as Mean ± SD, n = 11/group. Within each dose (0.83, 1.65 and 2.47 Gy), different symbols (**α**, **β**, **γ**) denote statistical significance (***p* < 0.05**) from 4^th^ to 14^th^ week of age. Abbreviations: TMD, tissue mineral density; Tt.Ar, cross-sectional area inside the periosteal envelope; Ct.Ar, cortical bone area; Ct.Th, cortical thickness; Ps.Pm, periosteum perimeter; Ec.Pm, endocortical perimeter; Ma.Ar, medullary area; Ecc, mean eccentricity.

Tukey’s post hoc multiple comparison tests revealed differences among different groups for the 14^th^ week scanning data (Table 4). 0.83 Gy group showed significant difference with the 1.65 Gy group for BMD, Tb.Th, Conn.Dn, and Tt.Ar parameters (Table 4). Comparing 0.83 Gy and 2.47 Gy groups, significant differences were found for BMD, Tb.Th, Tb.N, Tb.Sp, Conn.Dn, Ct.Th, and Ps.Pm parameters (Table 4). The 1.65 Gy group showed significant differences with the 2.47 Gy group for Tt.Ar parameter only (Table 4).

**Table 4:** ANOVA test with Tukey’s multiple comparisons for the trabecular and cortical bone structural properties of the irradiated rat tibiae for three radiation groups on the 14^th^ week.

		Statistical Comparison		
		Irradiated (right) tibia		
	ANOVA *p*-values	0.83 Gy -	0.83 Gy -	1.65 Gy -
		1.65 Gy	2.47 Gy	2.47 Gy
***Trabecular bone structural properties***				
BMD (gm/cm^3^)	**< 0.001**	Yes	Yes	No
BV/TV (%)	0.067	-	-	-
Tb.Th (mm)	**< 0.001**	Yes	Yes	No
Tb.N (mm^-1^)	**0.003**	No	Yes	No
Tb.Sp (mm)	**0.039**	No	Yes	No
Conn.Dn (mm^-3^)	**0.025**	Yes	Yes	No
***Cortical bone structural properties***				
TMD (gm.cm^-3^)	0.056	-	-	-
Tt.Ar (mm^2^)	**0.048**	Yes	No	Yes
Ct.Ar (mm^2^)	0.704	-	-	-
Ct.Th (mm)	**0.044**	No	Yes	No
Ps.Pm (mm)	**0.017**	No	Yes	No
Ec.Pm (mm)	0.421	-	-	-
Ma.Ar (mm^2^)	0.572	-	-	-
Ecc	0.302	-	-	-

The given *p*-values are the results of a one-way ANOVA comparing the bone structural properties of the irradiated tibiae on the 14^th^ week among three doses groups. A bold value indicates a significant difference at ***p* < 0.05**. The “Statistical Comparison” columns indicate whether the radiation groups were significantly different using Tukey’s post-hoc pairwise comparisons.

### Body weight

**Fig 8:**
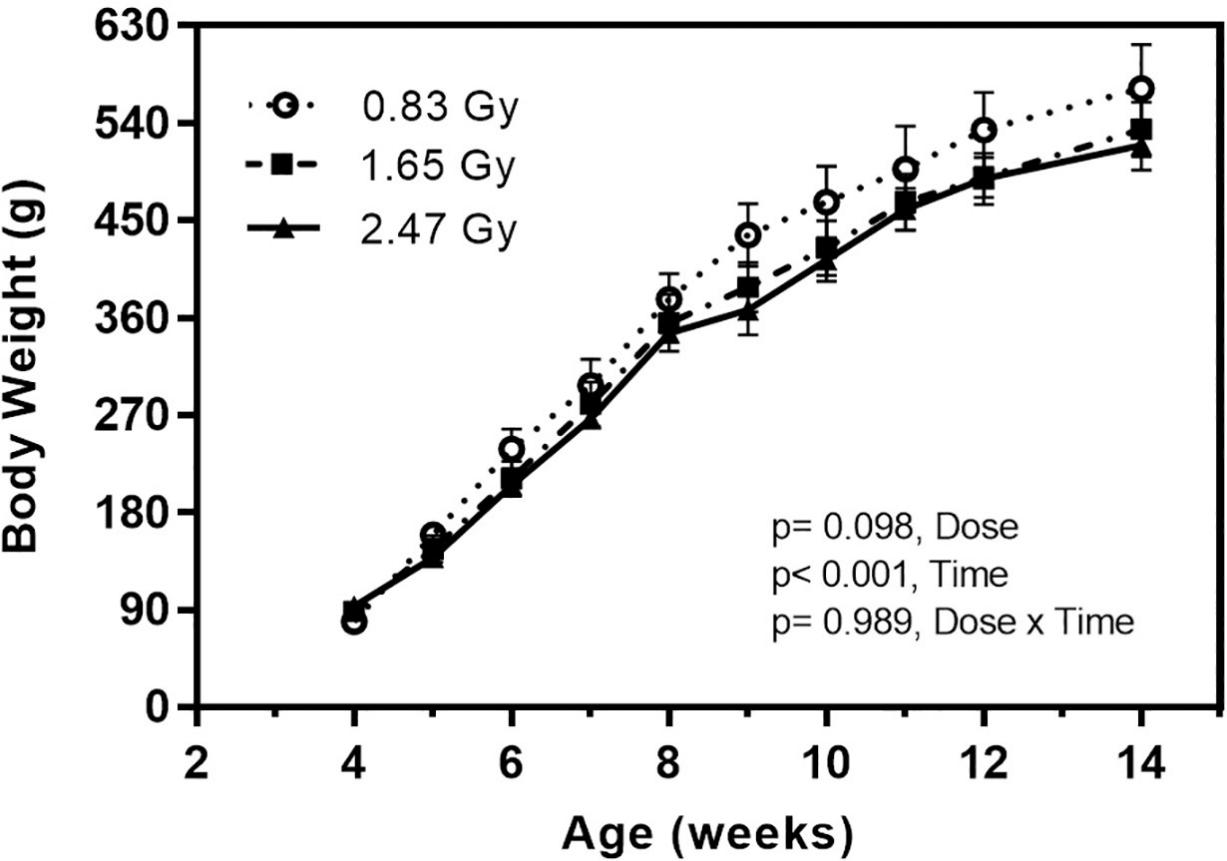
Body weight of male Sprague Dawley rats for three doses groups over the adolescent period. ANOVA test (general linear model) was performed to determine time effects, radiation dose, and their interaction on body weight. N = 11 rats per group (mean value ± SD).

Body weights were similar for rats of all groups at the beginning of the experiment (4^th^ week of age) (Fig 8). A time effect (weight gain) was observed in rats as they were in their growing phase. However, no effects of dose or dose/time interaction were found and no loss of hair was observed during the study period (Fig 8). At the end of the experiment, average body weights of 0.83, 1.65 and 2.47 Gy groups were 572.2 ± 40.8, 534.1 ± 25.5, and 519.2 ± 23.1 g, respectively.

### Bone marrow cells

Results showed no significant difference (*p* = 0.93) between percentage of unaffected bone marrow cells for control (93.2%) and irradiated tibiae (91.1%) at the 14^th^ week for 0.83 Gy group (Table 5). However, for 1.65 Gy group, a significant difference was observed (*p* = 0.04) between percentage of unaffected bone marrow cells for control (87.3%) and irradiated tibiae (71.6%) (Table 5). A significant difference was also observed (*p* = 0.02) between control (88.7%) and irradiated tibiae (70.8%) for the 2.47 Gy group (Table 5).

**Table 5:** Percentage of unaffected bone marrow cells for 0.83, 1.65 and 2.47 Gy radiation groups extracted from trypan blue test (mean value ± SD).

Radiation group	Tibiae	Unaffected bone marrow cells (%)	*p*-values
**0.83 Gy**	*Control*	93.20 ± 3.45	0.926
	*Irradiated*	91.11 ± 4.23	
**1.65 Gy**	*Control*	87.34 ± 7.37	**0.037**
	*Irradiated*	71.56 ± 9.27	
**2.47 Gy**	*Control*	88.67 ± 6.62	**0.021**
	*Irradiated*	70.84 ± 8.51	

Values are expressed as Mean ± SD. Both tibiae (n = 11 rats/group) were used for the analysis. A bold value indicates a significant difference (***p < 0*.*05***) between the control (left) and irradiated (right) tibiae for each radiation dose.

## Discussion

In this study, we investigated the effects of nine weeks *in vivo* scanning regime on the rat proximal tibiae under three different radiation doses. We used growing rats (n = 33), for which the right proximal tibia was irradiated while the left tibia was used as a non-radiated contralateral control. Bone growth, histomorphometry, morphology, and bone architecture during the growing period were assessed to identify the effects of repeated *in vivo* irradiation in the adolescent period. This study would optimally provide an effective radiation doses protocol, which would be “safe” to use for the growing rats. An effective radiation dose can be marked as “safe” if high-quality image sets can be acquired during the bone growing period without influencing the bone tissue health. We induced radiation doses with a higher frequency than generally used in bone investigation studies [43, 44], but similar to recent radiation effect investigation studies [24, 25]. Our adapted highest dose of radiation (2.47 Gy/scan) for 9 weeks is also within the limit of single dose of irradiation (2.5 Gy) for the tibial metaphysis of adult (10 and 14 months old) rabbits [20], where no significant alteration in bone formation was found. However, we investigated the growing animals (4^th^ to 14^th^ week of age) and our adapted radiation groups (0.83, 1.65 and 2.47 Gy) demonstrated mixed impacts on the bone microstructure during the study period.

### Radiation doses of 1.65 and 2.47 Gy adversely impacted tibial bone development during the adolescent growth period

Indeed, our results showed that these radiation doses reduced the hypertrophic and proliferative zone heights, which eventually inhibited bone growth rate of proximal tibiae. Both hypertrophic and proliferative cellular activities have an important impact on endochondral bone formation [45]. The main functions of the proliferative zone consist of matrix production and cellular proliferation [46]. Active cell replication takes place in this zone and chondrocytes are oriented in column formation along longitudinal bone growth [47]. The main functions of the hypertrophic zone include generating hypertrophic chondrocytes by terminal differentiation of the proliferative zone chondrocytes farthest from the epiphysis, preparing the matrix for calcification and to calcify the matrix [48]. Proliferative chondrocytes eventually increase in volume to generate the hypertrophic chondrocytes [49]. In the proliferative zone, cells undergo rapid replication [46]. In this region, chondrocyte divides, assume a flattened appearance, and become organized into columns parallel to the long axis of the bone [47]. Eventually, column elongation occurs through spatially coordinated cell division and rotational movements [47]. Hence, it is expected that any significant changes in these two zones will influence bone growth [45]. For the 0.83 Gy group, growth plate histomorphometry remained unaffected for the irradiated tibiae (Fig 5). However, for 1.65 and 2.47 Gy groups, a significant reduction in zone heights was observed for irradiated tibiae in the hypertrophic and proliferative area (Fig 5A and 5B). As a result, a significant reduction in overall bone growth rate for the irradiated tibiae was observed for both groups (Fig 2B). This decline in bone growth rate can be correlated with the reduction in proliferative and hypertrophic zone heights, which has also been observed in other studies [46, 50]. The average bone growth rates measured for both tibia in 0.83 Gy group are moreover similar to normal longitudinal bone growth rates observed in the rat tibia [35]. This indicates that the longitudinal bone growth was not affected by the 0.83 Gy radiation doses, which agree with other studies [24, 36], where also no effects of irradiation on the longitudinal bone growth were reported when using a similar radiation exposure level. The significantly reduced bone growth measured in 1.65 and 2.47 Gy groups also agree with the findings from other studies [27, 51, 52], where inhibition of bone growth was reported due to the effects of *in vivo* irradiation.

### Trabecular bone, together with bone marrow cells, were negatively affected when undergoing repeated radiation doses of 1.65 and 2.47 Gy

**Fig 9:**
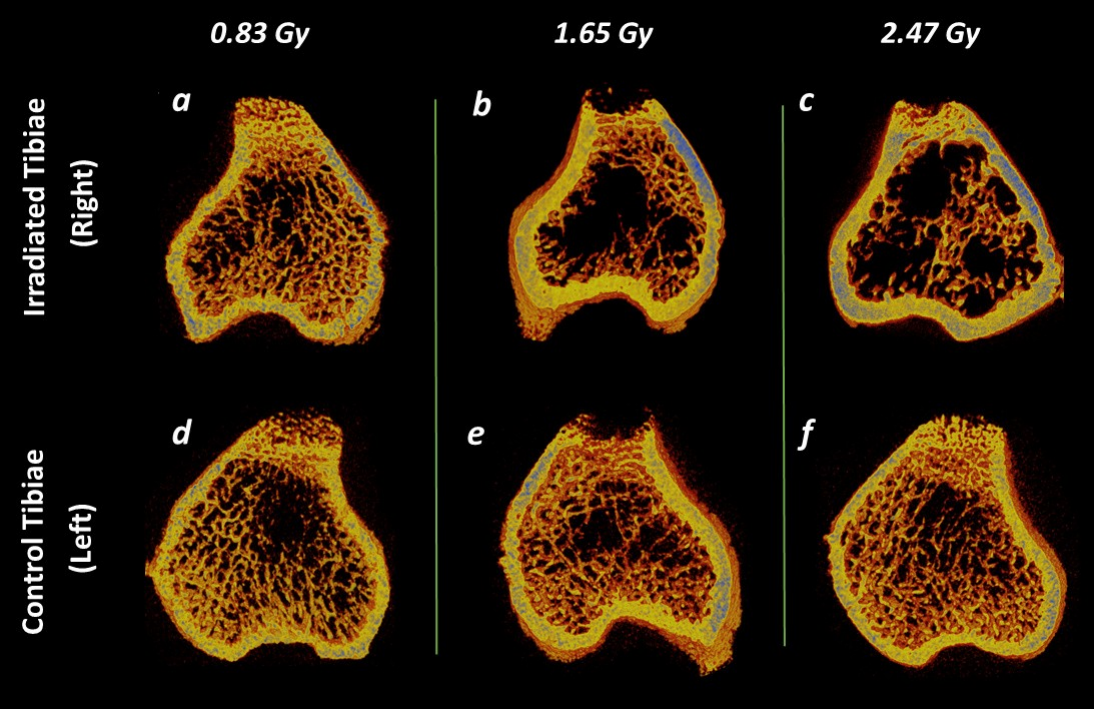
Trabecular and cortical bone representation after the 9-weekly *in vivo* micro-CT scans. (a—f) Representative 3D micro-CT images of metaphyseal bone structure of the irradiated (right) and non-irradiated control (left) tibiae at 14^th^ week of age after 0.83, 1.65 and 2.47 Gy radiation doses during the rat adolescent period. 3D micro-CT images within each radiation dose portray tibiae from the same rat, randomly selected to be representative of its respective dose group.

Our results showed that trabecular bone quantity and microstructure were adversely impacted for 1.65 and 2.47 Gy groups (Figs 6 and 9).

However, our findings showed no significant differences between the irradiated and contralateral tibiae for the trabecular bone microarchitectures for 0.83 Gy group of rats (Fig 6). Our findings are supportive of a study using adult rats [30] (12 weeks old), where no radiation effects (0.60 Gy) were found on the proximal tibiae after a 3-month study period with monthly scanning regime. In another study [24], adult Wister rats (30 weeks old) underwent 8 weeks *in vivo* tibial scanning under doses of 939 mGy per scan, but the bone structural measurements remained unaffected. However, our findings showed significant effects on the irradiated tibiae for 1.65 and 2.47 Gy groups.

Our repeated weekly *in vivo* irradiation resulted in a lower BMD in the irradiated tibia only for 1.65 and 2.47 Gy groups (Fig 6). From the longitudinal data, it can be observed that the 0.83 and 2.47 Gy group showed respectively a significant increase and decrease for the irradiated tibia in the BMD value from 4^th^ to 14^th^ week period (Table 2). In general, bone mineral content tends to increase at the young age for healthy bone [53]. Also, in the adolescent period, soft tissue thickness of the proximal tibia increases due to the bone growth in this period [54]. It might be possible that for the 1.65 and 2.47 Gy group, the radiation doses affected the proximal tibial thickness by increasing the osteoclastic activity during the irradiation process [55]. This phenomenon might have triggered the significant decrease in BMD in the irradiated tibiae for these groups (Figs 6 and 9). This reasoning is supported by another study, where the effect of radiation was assessed in the spine and the hip on 49 radiology and 40 non-exposed workers over ten years period [56]. A significant decrease in BMD was found among the workers who were exposed to the radiation. Also, other studies irradiating mice with 1–2 Gy doses reported a lower BMD after 12 weeks of post-irradiation [52, 57, 58].

An increase in BV/TV is often correlated with a rise of BMD for normal bone growth [59], which indicates a higher bone quality. This normal bone development phenomena can be observed for the 0.83 Gy group as the BV/TV values increased significantly during the adolescent period (Table 2). For the 0.83 and 1.65 Gy group, no significant difference was found between the contralateral tibiae on the 14^th^ week (Fig 6). However, BV/TV values decreased significantly compared to the control ones for the 2.47 Gy group (Fig 6), which could be associated with the diminishing trend observed earlier in the longitudinal BMD values for the same group. Our findings are supported by a study where a weekly radiation dose of 0.846 Gy over 5 weeks resulted in a decreased BV/TV in adult mice (12-week old) [25]. Also, another study reported a 30% loss in 10-week old mice BV/TV after performing three 0.776 Gy dose scans separated by 2-week intervals [9].

As for Tb.Th, Tb.N, and Tb.Sp, no difference was found between the irradiated and control tibiae for the 0.83 Gy group (Fig 6), while Tb.Th and Tb.N were significantly increased in the growing period for this group (Table 2). The increment of Tb.Th during the growing period indicates normal bone growth process [24, 60, 61]. Moreover, the observed increase in Tb.N is associated with the concomitant increase in BV/TV for the young age period [62]. These findings agree with results from other radiation effects investigation studies using 30 weeks old rats (0.60 Gy) [24], and 17 weeks old ovariectomized mice (1.30 Gy) [23]. In both of these studies, Tb.N, Tb.Th and Tb.Sp remained unaffected. However, for the 1.65 and 2.47 Gy groups, Tb.Th and Tb.N values were significantly lower and the Tb.Sp values were significantly higher in the irradiated tibiae (Fig 6). From the longitudinal data, a significant decrease for Tb.Th and a significant increase in Tb.N and Tb.Sp were observed for 1.65 Gy group (Table 2), whereas the 2.47 Gy group showed a significant decrease for Tb.Th and an increase for Tb.Sp only in the scanning period (Table 2). These phenomena indicate the occurrence of a radiation-induced bone loss through a decreased connectivity and a gradual thinning of the trabecular structure (Fig 9). Our data are supportive of previous findings from a mice study where a 5–6 Gy radiation exposure for 3 days and 14 days resulted in decreased Tb.Th, Tb.N, and an increased Tb.Sp [63]. Another mice study using 0.846 Gy radiation dose for 5 weekly scans at 2 weeks interval reported a lower Tb.Th, Tb.N, and a higher Tb.Sp [25].

As for Conn.Dn, no significant difference was observed between the irradiated and control tibiae at the 14^th^ week for all three groups (Fig 6). However, a significant decrease was observed from the longitudinal data for both 0.83 and 1.65 Gy groups (Table 2). Connectivity density is vital in the maintenance of bone strength and trabecular connectivity is a fundamental property of 3D bone networks. As Conn.Dn provides a measure of unconnected trabeculae, this decrement could occur because the bone was still in the growing phase while the trabecular structure was changing with time. This observation also agrees well with the findings from other rat studies [24, 39], where Conn.Dn was also decreased with the age of the rats.

Also from ANOVA test with Tukey’s multiple comparisons, it has been observed that for trabecular bone, 0.83 Gy group showed significant differences with the 1.65 and 2.47 Gy groups for BMD, Tb.Th, Tb.N, Tb.Sp, and Conn.Dn, whereas, no significant differences for the trabecular bone microstructure were found between 1.65 and 2.47 Gy groups (Table 4). This indicates higher similarities of trabecular morphometric data between 1.65 and 2.47 Gy group compared with the 0.83 Gy group. This observation agrees with our morphometric findings as both of this group demonstrated similar adverse effects on the bone microarchitecture compared to the 0.83 Gy group (Fig 9). It has been reported that a radiation dose, if too high, can cause cell death, and the effects can be apparent within hours, days, or weeks after the exposure period [64]. Therefore, a two-week interval for the last scan was implemented in this study considering the possibility that the maximal radiation exposure effect could occur after the end of the exposure protocol [27, 28]. Bone marrow cells remained unaffected for 0.83 Gy group, which agrees with the conjecture based on CTDI [12, 22], and with a recent study [24] (30 weeks old rats, 600 mGy), where no cell damage due to radiation was reported. However, for the 1.65 and 2.47 Gy groups, significant differences were observed between control and irradiated tibiae. This phenomena confirm the negative impacts of 1.65 and 2.47 Gy doses on bone tissue health and can also be correlated with the detrimental effects observed by these doses on the trabecular structure found in our study [65].

### Cortical bone quantity and microstructure were slightly deteriorated under repeated radiation dose of 2.47 Gy

As opposed to trabecular bone, tested radiation doses had no profound effects on cortical bone microarchitecture. Indeed, for all three groups, there was no significant difference observed between the irradiated and the control tibia for TMD, Tt.Ar, Ct.Ar, Ps.Pm, Ec.Pm, Ma.Ar, and Ecc (Fig 7). From the longitudinal data, a significant increase was observed for Tt.Ar, Ct.Ar, Ps.Pm, Ec.Pm, Ma.Ar, and Ecc in all groups, whereas TMD and Ct.Th increased for 0.83 and 1.65 groups indicating the normal bone growing phenomenon (Table 3). However, the cortical thickness (Ct.Th) showed a significant difference at the 14^th^ week scanning time point and decreased in the irradiated tibia compared to the control ones for the 2.47 Gy group (Fig 6). It could be possible that the radiation dose might have affected more intensively the vascularization of the proximal tibia. Vascularization is essential for bone formation and bone remodeling, transporting nutrients and the oxygen supply and allowing endothelial cells to communicate with osteoprogenitors and osteoclasts [66]. Moreover, if this process gets affected, a potential bone tissue destruction can occur [67]. Another possible reason could be the redistribution of bone mass from the endosteal region to the sub-periosteal region of the tibia. If this redistribution happens, it generally results in reduced cortical thickness of the bone diaphysis [68]. Our findings can be confirmed from another study [69] where a single 80-Gy radiation exposure for the 8-week-old rat hind limbs substantially decreased the cortical thickness and created wide bone gaps in the bone microstructure. Also from ANOVA test with Tukey’s multiple comparisons, it has been observed that for cortical bone, 0.83 Gy group showed significant differences with the 1.65 and 2.47 Gy groups for Tt.Ar, Ct.Th, and Ps.Pm, whereas, significant difference for only Tt.Ar was found between 1.65 and 2.47 Gy groups (Table 4). This findings indicate the vulnerability of cortical bone microarchitecture under the radiation doses of 1.65 and 2.47 Gy group which is in agreement to our morphometric findings.

### Comparisons among protocols used in similar radiation studies and strengths of the current study

Differences found between our results and published studies might result from different factors. The positioning of the animal’s limb in the scanner bed can be considered a possible factor for discrepancies among the studies. Since the right proximal tibia (irradiated) was exposed to radiation for 9 weeks (from 4^th^ to 14^th^ week of age), the frequent stretching of the right tibiae might have induced an effect on the bone tissue microstructure. As the radiation chamber rotates around the object for scanning, the right tibia was always pulled away from the body and fixed on the Styrofoam holder with the masking tape during the scanning period. The contralateral tibia was folded along with the tail outside the Styrofoam holder. This stretching could make the rat put a reduced pressure on right tibia for a short period of time right after the scanning period, which could lead to bone loss [24]. However, as we have followed the same approach throughout the whole study, this effect (if any) should be similar for all the animals and hence the relative comparison allows to draw conclusions. Also, it was presumed that the left tibia remained unaffected during the scanning of the right tibia. Nevertheless, it is possible that systemic radiation effects have occurred and affected the left tibia [24]. However, as we only irradiated the proximal tibial portion, which covered a small segment compared to the whole body, these systemic effects are expected to be non-significant. Also, changes in the body weight were compared with the literature [70, 71] to check for any sudden weight loss and no anomalies were found. Using various animal models might also be another contributing factor for discrepancies. It has been observed from the literature that, for mice, the scanning time interval might be more critical than the radiation doses [72]. Also, in some cases, a similar amount of radiation exposure for both mice and rats have produced divergent results. Rat bone structure seems to be more resilient to the same amount of radiation exposure compared to the mice [23–25, 52, 57]. One possible reason could be the presence of larger and thicker skeletons in rats compared to mice, which might provide an additional absorbing capacity of the induced radiation for rats. Another reason for the discrepancies might be the age of the animals used in different studies. In most studies, an adult animal model has been used compared to our adolescent model [23, 24, 39]. Bone remodeling gets slower with aging [73], and bone turnover rate shifts towards bone resorption [74, 75]. As a result, the bone microstructure behavior is expected to be different in these studies compared to ours. Nonetheless, it remains difficult to make a comparison of our findings with other studies as none of them investigated the effects of *in vivo* micro-CT irradiation in a rat model during its growing period (4^th^ to 14^th^ week of age) [26]. Also, the scanning protocol, radiation doses, types of scanner, and animal positioning during scanning can contribute to differences in results between different animal studies.

Despite some limitations, our current study possesses a number of strengths. First, to authors’ best knowledge, this study is the first of its kind to investigate the effects of repeated *in vivo* micro-CT irradiation using an animal model during its entire growing period. Second, three different levels of radiation doses have been investigated using the same image reconstruction parameters to facilitate the comparison among results from different groups. Third, we scanned the non-radiated control legs (left tibiae) only at the end point (14^th^ week of age). This method of using an internal control decreases the use of extra animals for control and reduces the variability in the extracted data sets. Furthermore, results from the current study possess insightful information regarding bone microarchitecture during the bone development period, which would be useful to the bone and orthopedic research community.

## Conclusion

In conclusion, using 1.65 and 2.47 Gy doses might yield better image quality for bone tissue investigation but possess a high risk of altering the bone growing process in the rat adolescent period. Our results showed that, under radiation doses of 1.65 and 2.47 Gy, trabecular bone, together with bone marrow cells, as well as tibial bone development were adversely impacted. Also, cortical bone quantity and microstructure was slightly deteriorated under repeated radiation doses of 2.47 Gy. Hence, it appears from our results that 1.65 and 2.47 Gy doses affected significantly the bone marrow cells, histomorphometric and morphological parameters, and longitudinal bone growth of the immature rats. However, the 0.83 Gy radiation exposure did not affect the bone tissue structure for the growing rats. These findings can be used as a proof of concept for using the reasonable high-quality image acquisition under 0.83 Gy radiation doses during the entire growing period of rats without interfering with the bone development process. Our study also advances the knowledge on the evaluation of the radiation effects during the adolescent period of animal models in order to provide functional information for the design of future *in vivo* studies, in which the repeated radiation exposure is necessary and can induce additional impacts on the outcomes. Considering that the radiation damage also depends on other factors (scanning protocol, systemic effects, site-specificity), which are not micro-CT system specific, careful consideration should be adapted for future studies.

## Supporting information

S1 File Certification of animal care. (PDF)

S2 File The ARRIVE guidelines checklist. (PDF)

S3 File Calculation of radiation doses per scan. (PDF)
