# Supplementary material for: Can repeated in vivo micro-CT irradiation during adolescence alter bone microstructure, histomorphometry and longitudinal growth in a rodent model?
Source: PLoS One. 2018 Nov 15;13(11):e0207323. doi: 10.1371/journal.pone.0207323 (PMC6237372; doi:10.1371/journal.pone.0207323)
Supplement: S1 File — (PDF) [file pone.0207323.s001.pdf]

Centre de
Recherche du
CHU Sainte-Justine
*Le centre hospitalier
universitaire mère-enfant*

Pour l'amour des enfants

Université de Montréal

Le 22 juin 2015

Docteure Isabelle Villemure
Laboratoire LIS 3D
Local 4715
CHU Ste-Justine

Objet: **Projet intitulé :** Effets des radiations in vivo provenant de l'imagerie par micro-CT sur la croissance osseuse pubertaire.

Docteure,

Suite à la réunion du Comité institutionnel de bonnes pratiques animales en recherche tenue le 21 mai 2015, votre nouvelle demande a été évaluée, approuvée, suite aux modifications qui ont été apportées, et codifiée **sous le 600/Catégorie D.**

Par conséquent, et *conditionnellement à l'espace disponible à l'animalerie*, vous êtes autorisé par la Direction du Centre de recherche à débuter votre projet pour l'année en cours.

Nous désirons également vous souligner l'importance de toujours indiquer le bon numéro se référant au protocole en titre, dans toute correspondance.

Nous demeurons à votre disposition pour toute information complémentaire. Agréez, l'expression de nos sentiments les meilleurs.

Monique Charette

Monique Charette
Coordonnatrice du CIBPAR
MC/mc

pj Certificat de bons soins aux animaux

cc Irène Londono

3175, Côte-Sainte-Catherine
Montréal (Québec)
H3T 1C5
www.chu-sainte-justine.org

COMITÉ INSTITUTIONNEL DE BONNES PRATIQUES ANIMALES EN RECHERCHE
CERTIFICAT DE BONS SOINS AUX ANIMAUX

INSTITUTIONAL COMMITTEE FOR ANIMAL CARE IN RESEARCH
CERTIFICATION OF ANIMAL CARE

CHERCHEUR PRINCIPAL/PRINCIPAL INVESTIGATOR:

SERVICE/DEPT.: GRDMS/ÉPM **CHU SAINTE-JUSTINE (Centre de recherche)**

TITRE DE LA DEMANDE DE SUBVENTION/TITLE OF GRANT APPLICATION:
Biomechanical and microstructural evaluation of newly formed bone following static/dynamic bone growth modulation in an immature animal model.

Le Comité institutionnel de bonnes pratiques animales en recherche, ayant reçu le protocole relatif à la demande de subvention à **l'organisme :** ________CRSNG__________ ______________ pour le projet susmentionné, concernant le soin et le traitement des animaux, a examiné les méthodes d'expérimentation proposée. Le Comité atteste, conjointement avec le candidat, que les animaux utilisés aux fins de ces travaux seront traités conformément aux principes énoncés dans les publications intitulées "Utilisation des animaux de laboratoire/ Procédures standards" Fév. 91.

The Institutional Committee for Animal Care in Research, having received the protocol relevant to **agency's:** ____NSERC_______________ grant application to support the above-named project, on matters relating to animal care and treatment, examines the experimental procedures proposed. It certifies, with the applicant, that the care and treatment of animals used will be in accordance with the principles published in the Canadian Council on Animal Care's "Guide to the Care and Use of Experimental Animals" Feb. 91.

En outre, le Comité examine et approuve les points de la proposition qui ont trait au soin et au traitement des animaux, ainsi que les méthodes d'expérimentation proposées avant qu'elles ne soient entreprises par le candidat. Cette façon de procéder s'applique au cours de la période entière des travaux de recherche.

Furthermore, all matters arising from this proposal that relate to animal care and treatment, and all experimental procedures proposed for use with animals are reviewed and approved by the Committee before they are initiated or undertaken by the applicant. This review process is ongoing on a regular basis during the entire period that the research is being undertaken.

____
Candidat(e)/Applicant - Signature

08 JUIN 2015
____
Date d'approbation / Date of approval

____
Florina Moldovan, Présidente du Comité institutionnel de bonnes pratiques animales en recherche / Chairperson of the Institutional Committee for Animal Care in Research

L'institution (CHU Sainte-Justine) atteste qu'elle veillera à l'observation de ces lignes directrices./The institution (Ste. Justine Hospital) certifies that it will monitor adherence to these guidelines.

HÔPITAL SAINTE-JUSTINE/Centre de recherche, 3175 Chemin Côte Ste-Catherine, Montréal, Québec H3T 1C5 - Tél./Tel.: (514) 345-4691 FAX: (514) 345-4801

CIBPAR 2015
