# Supplementary material for: Can repeated in vivo micro-CT irradiation during adolescence alter bone microstructure, histomorphometry and longitudinal growth in a rodent model?
Source: PLoS One. 2018 Nov 15;13(11):e0207323. doi: 10.1371/journal.pone.0207323 (PMC6237372; doi:10.1371/journal.pone.0207323)
Supplement: S3 File — (PDF) [file pone.0207323.s003.pdf]

Calculation procedure for the applied radiation doses in three different groups of rats:

Radiation doses for the SkyScan 1176 has been calculated based on the "SkyScan 1176 *in vivo* scanning: X-ray dosimetry" report. A brief description of how the dose measurements have been performed and listed results from the report is provided here.

Method

The dose measurements were carried out using an UNFORS PS-2 patient skin dosimeter. Shielding was provided with acrylic plastic (PMMA) tubes of various wall thicknesses to simulate biological soft tissue.

Results

The calculated radiation absorbed dose and corresponding effective dose equivalent values are derived from the measured dose rates with several PMMA shielding depths, extrapolated where necessary by exponential curve-fitting. For approximation of the mean dose rate at different tissue diameters, the tissue at all depths is assumed to be a cylinder, and the dose rate at all tissue cylinder diameters averaged between the dose in air (zero depth) and the dose at the cylinder center (half diameter).

Scan doses for maximal image quality scan settings: For the scanning settings of 65 kV, 384 µA x-ray - full power and 1mm Al filter, the local absorbed dose rate for *in vivo* rat tibial scanning is reported by Bruker to be 148.3 mGy/min [ref: SkyScan 1176 *in vivo* scanning: X-ray dosimetry]

So, for our experiment, the doses per scan calculation is as follows:

Group 1: Scanning time $= 5\,\mathrm{min}\,34\ sec = \left\{5 + \left(\frac{34}{60}\right)\right\}\mathrm{min} = 5.567\ min$

So, the radiation doses per scan $= Scanning\ time\ x\ dose\ rate$

$$= (5.567\ x\ 148.3)\ mGy = 825.59\ mGy = \mathbf{0.83\ Gy}$$

Group 2: Scanning time $= 11\,\mathrm{min}\,9\ sec = \left\{11 + \left(\frac{9}{60}\right)\right\}\mathrm{min} = 11.15\ min$

So, the radiation doses per scan $= Scanning\ time\ x\ dose\ rate$

$$= (11.15\ x\ 148.3)\ mGy = 1653.55\ mGy = \mathbf{1.65\ Gy}$$

Group 3: Scanning time $= 16\,\mathrm{min}\,39\ sec = \left\{16 + \left(\frac{39}{60}\right)\right\}\mathrm{min} = 16.65\ min$

So, the radiation doses per scan $= Scanning\ time\ x\ dose\ rate$

$$= (16.65\ x\ 148.3)\ mGy = 2469.19\ mGy = \mathbf{2.47\ Gy}$$
